# Decrease in membrane phospholipids unsaturation correlates with myocardial diastolic dysfunction

**DOI:** 10.1371/journal.pone.0208396

**Published:** 2018-12-11

**Authors:** Tsunehisa Yamamoto, Jin Endo, Masaharu Kataoka, Tomohiro Matsuhashi, Yoshinori Katsumata, Kohsuke Shirakawa, Naohiro Yoshida, Sarasa Isobe, Hidenori Moriyama, Shinichi Goto, Kaoru Yamashita, Hiroki Nakanishi, Yuta Shimanaka, Nozomu Kono, Ken Shinmura, Hiroyuki Arai, Keiichi Fukuda, Motoaki Sano

**Affiliations:** 1 Department of Cardiology, Keio University School of Medicine, Tokyo, Japan; 2 Japan Science and Technology Agency, Tokyo, Japan; 3 Department of Endocrinology and Hypertension, Tokyo Women’s Medical University, Tokyo, Japan; 4 Research Center for Biosignal, Akita University, Akita, Japan; 5 Graduate School of Pharmaceutical Sciences, Tokyo University, Tokyo, Japan; 6 Department of General Medicine, Hyogo College of Medicine, Hyogo, Japan; Niigata Daigaku, JAPAN

## Abstract

Increase in saturated fatty acid (SFA) content in membrane phospholipids dramatically affects membrane properties and cellular functioning. We sought to determine whether exogenous SFA from the diet directly affects the degree of membrane phospholipid unsaturation in adult hearts and if these changes correlate with contractile dysfunction. Although both SFA-rich high fat diets (HFDs) and monounsaturated FA (MUFA)-rich HFDs cause the same degree of activation of myocardial FA uptake, triglyceride turnover, and mitochondrial FA oxidation and accumulation of toxic lipid intermediates, the former induced more severe diastolic dysfunction than the latter, which was accompanied with a decrease in membrane phospholipid unsaturation, induction of unfolded protein response (UPR), and a decrease in the expression of Sirt1 and stearoyl-CoA desaturase-1 (SCD1), catalyzing the conversion of SFA to MUFA. When the SFA supply in the heart overwhelms the cellular capacity to use it for energy, excess exogenous SFA channels to membrane phospholipids, leading to UPR induction, and development of diastolic dysfunction.

## Introduction

Accumulation of excess lipids in non-adipose tissues, due to overeating or a sedentary lifestyle, leads to cell dysfunction or death. This phenomenon, known as lipotoxicity, plays an important role in the pathogenesis of heart failure in humans [1–4].

In animal experiments, systemic metabolic alterations that result in cardiac lipid accumulation are associated with cardiac dysfunction [5]. Moreover, cardiac lipid accumulation in mice isolated from systemic metabolic disturbance indicates that mismatch between lipid import and use in the heart is sufficient to induce cardiac dysfunction [6]. For example, cardiomyocyte-specific increase in fatty acid (FA) uptake in mice with cardiac-specific overexpression of long chain acyl-CoA synthetase 1 [7], lipoprotein lipase [8], or fatty acid transporter 1 [9] are sufficient to induce cardiomyocyte dysfunction and/or death, leading to left ventricular (LV) dysfunction. Mice with cardiac-restricted overexpression of the FA-activated nuclear receptor, peroxisome proliferator-activated receptor alpha (PPARα), exhibit myocyte lipid accumulation and cardiac dysfunction [10], which are rescued by inhibition of FA uptake through deletion of CD36 [11] or lipoprotein lipase [12]. The proposal mechanisms responsible for cardiac dysfunction due to mismatch between lipid import and use in the heart include accumulation of toxic lipid intermediates within cardiac myocytes, generation of harmful reactive oxygen species due to increased mitochondrial flux, and reliance on FA oxidation for ATP production, which results in higher mitochondrial oxygen consumption costs compared with glycolysis and glucose oxidation [10].

Studies using cultured cells to model lipotoxic response have helped elucidate the mechanism involved in the differential effects of SFA and MUFA on cellular function [13–18]. Palmitate (SFA) induces cell death, while oleate (MUFA) did not and reduced the palmitate-induced cell death when the two were given in concert. Studies using primary cardiomyocytes cultures from embryonic chicks, neonatal rats, or H9C2 cardiomyocytes showed that incubation with excess palmitate caused the loss of mitochondrial membrane potential, mitochondrial swelling and cytochrome c release [19, 20]. These phenomena may be preceded by several events including decreased synthesis of cardiolipin in mitochondria [21], increased de novo ceramide formation [22], increased generation of reactive oxygen species [23, 24], JNK activation [25], induction of unfolded protein response (UPR) [23], shifts in the endoplasmic reticulum (ER), and mitochondrial dynamics towards fission and away from fusion [17, 26].

Several observations have suggested that the FA composition of membrane phospholipids can be affected by exogenous FAs from the diet or by altered activity of lipid-metabolizing enzymes such as stearoyl-CoA desaturase 1 (SCD1). Palmitate is rapidly incorporated into the lipid component of ER, which impaired ER structure and integrity in CHO cells [27]. SCD1, which is expressed on the ER membrane plays a protective role in palmitate-induced toxicity [28–30]. This evidence suggested that the ER may play a proximal role in SFA-induced lipotoxicity by changing the FA composition of membrane phospholipids.

The adult heart consumes more fat than any other organ in the body. About two thirds of acetyl CoA formation is covered by oxidative phosphorylation of FA in mitochondria. It should be immediately apparent that, unlike cancer cells or immature cardiomyocytes, the large energy demands of the functioning cardiomyocyte would not channel most exogenous FA toward lipid membranes under unstressed conditions. According to current understanding, a large fraction of the exogenous long chain FAs is esterified into the triglyceride (TG) pool upon entry into the cardiomyocyte and then later de-esterified for release and entry into β-oxidation in the mitochondria [31]. However, when SFA overload in the heart overwhelms the cellular capacity to use it for energy or to store FA as TG, excess FA may channel to membrane phospholipids. In this situation, the FA composition of membrane phospholipids is likely to be affected by diet even in fully differentiated adult cardiomyocytes.

In this study, by comparing the hearts of mice fed high fat diets with different SFA and MUFA balances, we sought to determine whether exogenous FAs from the diet directly affect the degree of membrane phospholipid unsaturation and activities of lipid metabolizing enzymes in the cardiomyocytes of adult functioning hearts. Then, we asked whether these changes correlate with contractile dysfunction of fat overfed hearts.

## Materials and methods

### Animal work

C57BL/6J male mice were housed under a 12-hour light/12-hour dark cycle and allowed free access to food. Mice were fed either a control diet (D12450J, 10kcal% fat, Research Diets, see Table 1), a high lard diet (HLD) (D12492, 60kcal% fat, Research Diets, see Table 1), or a high olive-oil diet (HOD) (D01112603, 60kcal% fat, Research Diets, see Table 1). Mice were fed with either an HLD or HOD starting at 4 weeks of age or kept on a control diet. We analyzed mice at 20 weeks of age. Euthanasia was performed by Pentobarbital intraperitoneal overdose (300mg/kg).

**Table 1:** Diet composition.

Food name	Control diet (CD)		High lard diet (HLD)		High olive oil diet (HOD)	
Product #	D12450J		D12492		D01112603	
	gm %	kcal %	gm %	kcal %	gm %	kcal %
Protein	19.2	20.0	26.2	20	26.2	20
Carbohydrate	67.3	70.0	26.3	20.1	26.3	20.1
Fat	4.3	10.0	34.9	59.9	34.9	59.9
Total		100.0		100		100
kcal/gram	3.85		5.24		5.24	
Ingredient						
	gm	kcal	gm	kcal	gm	kcal
Total	1055.05	4057	773.85	4057	773.85	4057
Casein, 80 Mesh	200	800	200	800	200	800
L-Cystine	3	12	3	12	3	12
Corn Starch	506.2	2024.8	0	0	0	0
Maltodextrin 10	125	500	125	500	125	500
Sucrose	68.8	275.2	68.8	275.2	68.8	275.2
Cellulose, BW200	50	0	50	0	50	0
Soybean Oil	25	225	25	225	25	225
Lard	20	180	245	2205	0	0
Olive Oil	0	0	0	0	245	2205
Saturated fat (gm%)	23.5		32.2		14.2	
Monounsaturated fat (gm%)	29.7		35.9		67.7	
Polyunsaturated fat (gm%)	46.8		31.9		18.1	
Mineral Mix S10026	10	0	10	0	10	0
DiCalcium Phosphate	13	0	13	0	13	0
Calcium Carbonate	5.5	0	5.5	0	5.5	0
Potassium Citrate, 1H2O	16.5	0	16.5	0	16.5	0
Vitamin Mix V10001	10	40	10	40	10	40
Choline Bitartrate	2	0	2	0	2	0

### Quantification of fatty acid composition

Lipids from hearts, cardiomyocytes and serum were extracted by the method of Bligh and Dyer [32]. Phospholipids were isolated by application of the lipid extract to a column of silica coupled with aminopropyl groups (InertSep NH2, GL sciences, Tokyo, Japan). Isolated phospholipids were methylated with 2.5% H_2_SO_4_ in methanol. The resulting fatty acid methyl esters were then extracted with hexane and subjected to gas chromatography-mass spectrometry (GC-MS) analysis. GC-MS analysis was performed using an Agilent 7890A-5975C GC-MS network system (Agilent Technologies, Wilmington, DE) equipped with a DB-23 capillary column (60nm × 250μL × 0.15μm; Agilent Technologies.) The oven temperature program was as follows: the initial temperature was 50°C for 1min then raised to 175°C at 25°C/min, to 235°C at 5°C/min, and held for 5 min. The injector and detector temperatures were both set at 250°C.

### Quantification of ceramide in hearts

LC-electrospray ionization-MS/MS analysis was performed with an UltiMate 3000 LC system (Thermo-Fisher Scientific) equipped with an HTC PAL autosampler (CTC Analytics). A 10 μL aliquot of the lipid samples was injected and the lipids were separated on a Waters X-Bridge C18 column (3.5 μm, 150 mm × 1.0 mm i.d.) at room temperature (25°C) using a gradient solvent system as follows: mobile phase A (isopropanol/methanol/water (5/1/4 v/v/v) supplemented with 5 mM ammonium formate and 0.05% ammonium hydroxide)/mobile phase B (isopropanol supplemented with 5 mM ammonium formate and 0.05% ammonium hydroxide) ratios of 70%/30% (0 min), 50%/50% (2 min), 20%/80% (13 min), 5%/95% (15–30 min), 95%/5% (31–35 min) and 70%/30% (35–45 min). Flow rate was 20 μL/min. Ceramide was measured by selected reaction monitoring (SRM) in positive ion mode with a triple-stage quadrupole mass spectrometer (TSQ Vantage AM, Thermo-Fisher Scientific).

### Quantification of diacylglycerol in hearts

LC-electrospray ionization-MS/MS analysis was performed with a Nexera X2 system (two LC-30AD pumps, a CTO-20AC column oven unit, a SIL-30AC autosampler, a SBM-20A controller) (SHIMADZU). A 10 μL aliquot of the lipid samples was injected and the lipids were separated on a Waters CORTECS UPLC C18 column (1.6 μm, 150 mm × 2.1 mm i.d.) at room temperature (40°C) using a gradient solvent system as follows: mobile phase A (acetonitrile/methanol/water (9/9/2 v/v/v) supplemented with 5 mM ammonium formate and 0.05% ammonium hydroxide)/mobile phase B (isopropanol supplemented with 5 mM ammonium formate and 0.05% ammonium hydroxide) ratios of 90%/10% (0–5 min), 60%/40% (5–9 min), 45%/55% (9–17 min), 40%/60% (17–25 min), 40%/60% (25–38 min), 30%/70% (38–42 min), 25%/75% (42–72 min), 10%/90% (72–95 min), 10%/90% (95–107 min), 90%/10% (107–108 min) and 90%/10% (108–120 min). Flow rate was 50 μL/min. Diacylglycerol was measured by multiple reaction monitoring (MRM) in positive ion mode with a triple-stage quadrupole mass spectrometer (LCMS-8040, SHIMADZU).

### Quantification of triacylglycerol in hearts

Quantification of triacylglycerol in hearts was performed using a Triglyceride Assay Kit (ab65336, Abcam, Tokyo, Japan) following the manufacturer’s instructions.

### Hemodynamic pressure-volume measurements

Mice were anesthetized by isoflurane. A 1.4F pressure-conductance catheter (SPR-839; Millar instruments) was inserted into the right carotid artery and advanced into the ascending aorta. After recording aortic blood pressure, the catheter was advanced through the aortic valve into the LV where PV signals were continuously obtained (MRVS ultra, Millar instruments) and recorded in digital form (MPVS PL3508 PowerLab 8/35, ADInstruments) at the acquisition rate of 2kHz for later offline analysis (LabChart 8 pro, ADInstruments). Animals were allowed to stabilize for 5 min, then baseline load-independent parameters of systolic and diastolic function, including LV end-systolic pressure (ESP), LV end-diastolic pressure (EDP), cardiac output (CO) were measured and averaged from 10 consecutive beats. After baseline measurements, transient occlusion of the inferior vena cava was performed and used to calculate multibeat-derived, load-dependent measures of cardiac systolic and diastolic functions: linear end-systolic pressure-volume relationship (ESPVR), calculated as ESP = end-systolic elastance (Ees) × ESV + V_0_, was used for the evaluation of cardiac contractility, whereas exponential end-diastolic pressure-volume relationship (EDPVR), calculated as EDP = α × exp^β×EDV^, was implemented in assessing end-diastolic stiffness.

### Echocardiography

At the age of 20 weeks old, mice were anesthetized with a 1.5% isoflurane inhalation, and then were anchored to a positioning platform in the supine position. Short-axis echocardiographic measurements were made using the Vevo 660 system (Visual Sonics) with 6000 series real-time microvisualization scanhead probe [33]. The LV internal end-systolic and end-diastolic diameters (LVESD and LVEDD, respectively) were measured using the leading-edge convention adopted by the American Society of Echocardiography. LV fractional shortening (FS) was calculated according to the formula: FS (%) = ([LVEDD−LVESD]/ LVEDD) × 100. Heart rate did not differ significantly among the groups during the echocardiographic assessments.

### Glucose and insulin tolerance tests

We performed glucose tolerance (oral administration of 1.5 g/kg of glucose, after 16h of fasting) and insulin tolerance (administration of 0.75–2 U/kg of insulin intraperitoneally, after 4h of fasting) tests to assess glucose intolerance and insulin resistance as described [34].

### Histological analysis

Mice were anesthetized and sacrificed. Hearts were then immediately perfused with PBS, fixed with 10% formalin neural buffer solution, and embedded in paraffin. Hematoxylin & Eosin staining and Azan staining were performed following standard procedures. To evaluate the fibrotic area, short axis sections at the mid LV level were stained with Azan and analyzed quantitatively against control reference sections using BZ-II Analyzer software (Keyence) on a BIOREVO BZ-9000 microscope (Keyence). The number of pixels with the predetermined level of blue tone were counted in each section, and then automatically converted into dimensions. The myocyte cross-sectional area was measured from images captured from WGA-stained sections. The outlines of cardiomyocytes were traced in each section using ImageJ software.

### Evaluation of apoptosis

DNA fragmentation was detected with the use of TUNEL. Nuclear density was determined by manual counting of DAPI-stained nuclei in 8 to 10 fields for each sample, and the number of TUNEL-positive nuclei was counted by examining the same fields.

### Assay of Sirt1 activity

Sirt1 deacetylase activity was determined with the SIRT1 Fluorescent Activity Assay/Drug Discovery kit (Cat. No. BML-AK555, Enzo Life Science International) based on Fluor de Lys-SIRT1 substrate peptide. Protein extracts (10 μg) from mouse hearts were incubated with the fluorogenic acetylated peptide substrate. The reaction was carried out at 37°C for 1h, and the fluorescent signal was measured at 360nm excitation and 460nm emission on a fluorescence plate reader (SynergyHTX, BioTek).

### Western blotting

Standard SDS-PAGE Western immunoblotting techniques were used to assess the expression of SCD1, Sirt1, and GAPDH. Equal amounts of the total proteins of hearts were subjected to SDS-PAGE. The primary antibodies used in the present study were anti-phosphoAMPKα(Th172) (#4188), anti-AMPK (#2532), anti-Sirt1 (#8649), anti-SCD1 (#2794), anti-GAPDH (#2118) (Cell Signaling Technology, Danvers, MA, USA).

### Cell culture

Primary cultures of neonatal rat cardiomyocytes were prepared as described as previously [35]. In brief, neonatal ventricular myocytes from 1- to 2-day-old Sprague-Dawley rats were sacrificed by decapitation and hearts were immediately removed and subjected to Percoll gradient centrifugation and differential plating to enrich the cardiac myocyte population and to deplete non-myocytes. Cardiomyocytes were cultured in a mixture of Dulbecco’s modified Eagle’s medium (DMEM) and M199 with 10% fetal bovine serum (FBS; Biowest, Nuaille, France).

### Fatty acid stimulation

Fatty acids were dissolved in ethanol, and 100 mmol/L stock solutions were stored at −20°C. These stock solutions were combined with fatty acid-free bovine serum albumin (BSA, 10% concentration) at a molecular ratio of 3:1 (fatty acid:albumin). The indicated concentrations of fatty acids were prepared in serum-free medium. Palmitate (Cat. No. P0500) and Oleate (Cat. No. O1008) were obtained from Sigma Aldrich (St Louis, MO, USA). Fatty acid-free bovine serum medium (Cat. No. 011–15144) was obtained from Wako Pure Chemical Industries (Osaka Tokyo).

### Quantitative real-time PCR

For quantitative real-time polymerase chain reaction (PCR), total RNA samples from hearts were prepared using Trizol reagent (Invitrogen). Samples of total RNA (0.1–0.3μg) were reverse-transcribed using an RNA PCR kit (Takara Biotechnology, Otsu, Shiga, Japan), and the resulting cDNA was used as a PCR template. RNA levels were then determined by real-time PCR using the ABI PRISM7700 Sequence Detector (Applied Biosystems). Predesigned gene-specific primer and probe sets (TaqMan Gene Expression Assays) were also used, and *Gapdh* was amplified as an internal control. Relative gene expression levels (ie, the amount of target gene normalized against that of the internal control) were calculated using the comparative cycle threshold (Ct) method as 2^-ΔΔCt^. The primer sequences for genes are listed in S1 and S2 Tables.

### Quantitative real-time PCR for microRNA

Expression levels of microRNA(miR)-195 and miR-451 were measured using TaqMan MicroRNA Assays (Applied Biosystems) and TaqMan MicroRNA Reverse Transcription Kit (PN4366596, Applied Biosystems) in accordance with the manufacturer’s manual. Expression levels of miRs were normalized by U6 small nuclear RNA and calculated by the 2^-ΔΔCt^ method. The assay IDs were as follows: has-miR-195(000494), mmu-miR-451(001141), U6 snRNA(001973).

### Statistical analysis

Data are presented as mean ± SEM. The statistical significance of differences between the 2 groups was determined using a 2-tailed Student’s T test. Differences among multiple groups were compared using ANOVA followed by post hoc Tukey–Kramer tests. A value of *P* < 0.05 was considered statistically significant.

### Study approval

All procedures in the present study conformed to the principles outlined in the guide for the care and use of laboratory animals published by the US National Institutes of Health (NIH) and were approved by the International Animal Care and Use Committee of Keio University School of Medicine.

## Results

### HFD-induced diastolic dysfunction was correlated with the degree of fatty acid saturation in membrane phospholipids

**Fig 1:**
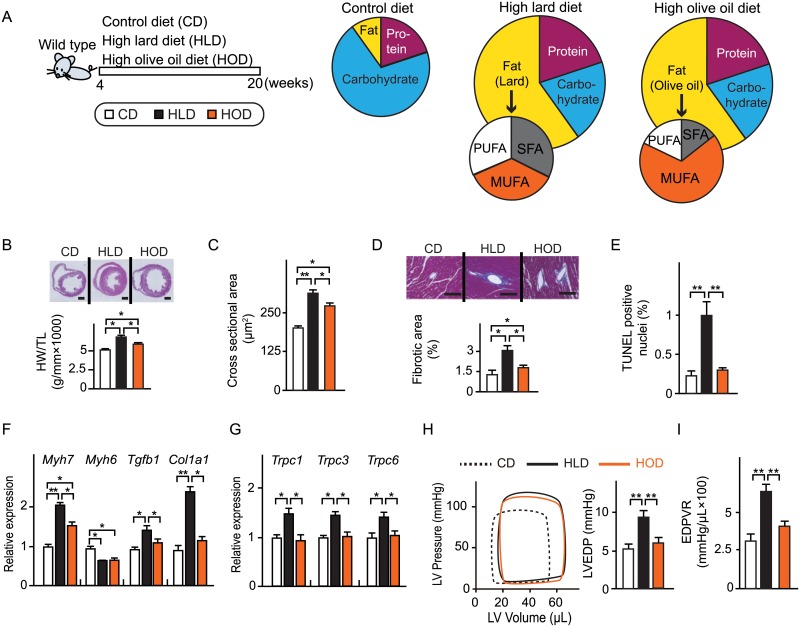
SFA-rich HFD caused cardiac hypertrophy, fibrosis, and diastolic dysfunction. (A) Schema of HFD feeding protocol. Mice were fed either a high lard diet (HLD, rich in SFA), a high olive-oil diet (HOD, rich in MUFA), or a control diet (CD) from 4 weeks to 20 weeks of age. (B) Top, representative low-magnification views of hematoxylin and eosin staining of the heart sections. Scale bars: 1 mm. Bottom, heart weight (HW) normalized by tibial length (TL). (n = 6). (C) Quantification of cardiomyocyte surface area. (n = 3). (D) Top, representative image of azan staining of the heart sections. Scale bars: 100 μm. Bottom, quantification of the fibrotic area. (n = 3). (E) Quantification of TUNEL-positive cardiomyocytes. (n = 3). (F) Relative expression of hypertrophy-associated and fibrosis-associated genes. (n = 6). (G) Relative expression of TRPC channels. (n = 6). (H) Left, representative pressure–volume curve. Right, LV end-diastolic pressure (LVEDP). (n = 10). (I) End-diastolic pressure–volume relationship (EDPVR). (n = 10). Data were presented as mean ± SEM. * *P* < 0.05, ** *P* < 0.01, by ANOVA followed by post hoc Tukey–Kramer tests. See also Tables 1 and 2.

Four-week-old male mice were randomly divided into three groups and fed with one of two different HFDs (60 Kcal% fat): a lard-based HFD (high lard diet: HLD, 32.2% of total fat is SFA, 35.9% MUFA), an olive oil-based HFD (high olive oil diet: HOD, 14.2% of total fat is SFA, 67.7% MUFA), or a control diet (CD) (10 Kcal% fat). Mice were analyzed at 20 weeks of age. (Fig 1A, Table 1). Lipidomic analysis of the serum FA composition revealed that SFA (palmitate C16:0 and stearate C18:0) were dominantly increased in mice fed a HLD, while MUFA (oleate C18:1) was dominantly increased in mice fed a HOD (S1B Fig). Both HFD groups developed the same levels of visceral adiposity as indicated by an increase in body weight, epididymal fat weight, and liver weight (S1C–S1E Fig). However, the HLD impaired glucose tolerance and insulin sensitivity more seriously than the HOD (S1F Fig).

We then compared the pathological and functional changes in the heart between the HLD and HOD groups. Cardiac hypertrophy and fibrosis, as indicated by increased heart weight normalized by tibial length, increase in cross sectional area (Fig 1B and 1C), and increase in fibrotic area (Fig 1D) in the HLD group, were more prominent than in the HOD group. The percentage of TUNEL-positive cardiomyocytes was higher in the HLD group than in the HOD group (Fig 1E). In accordance with this, the reactivation of fetal cardiac genes for contractile proteins and the induction of pro-fibrotic genes were observed (Fig 1F). Moreover, the transient receptor potential (TRP) gene family genes were more pronounced (Fig 1G). In terms of cardiac function, there was no difference in LV systolic function, indicated by fractional shortening (%FS), and the slope of the end-systolic pressure-volume relationship (ESPVR) in both HFD groups. However, the index of diastolic dysfunction, quantified by LV end-diastolic pressure (LVEDP) and the slope of the end-diastolic pressure-volume relationship (EDPVR), were significantly increased only in the HLD group (Fig 1H and 1I, Table 2). Myocardial afterload increased to the same degree in both HOD-fed hearts and HLD-fed hearts, as indicated by end-systolic pressure (ESP) and intra-aortic systolic pressure (Table 2).

**Table 2:** Comparison of cardiac function.

Food type	CD	HLD	HOD
N	10	10	10
Heart Rate (beats/min)	500 ± 5	503 ± 6	498 ± 8
CO (ml/min)	20 ± 2	21 ± 3	21 ± 4
ESP (mmHg)	93 ± 3	109 ± 2 *	106 ± 2 *
EDP (mmHg)	5.2 ± 0.5	9.2 ± 0.5 **	5.4 ± 0.3 ^##^
ESPVR (×100 mmHg/μL)	201 ± 23	203 ± 14	199 ± 18
EDPVR (×100 mmHg/μL)	3.4 ± 0.3	6.9 ± 0.3 **	4.2 ± 0.4 ^##^
IVS (×10 mm)	8.1 ± 0.3	9.6 ± 0.3 **	8.8 ± 0.2 *^#^
PW (×10 mm)	8.0 ± 0.2	9.5 ± 0.3 **	8.7 ± 0.3 *^#^
LVEDD (×10 mm)	35.8 ± 0.4	38.8 ± 0.4 *	37.1 ± 0.4 *^#^
LVESD (×10 mm)	20.3 ± 0.4	21.5 ± 0.6	20.8 ± 0.6
FS (%)	43 ± 1	45 ± 1	42 ± 2
EF (%)	69 ± 2	71 ± 3	68 ± 2

CD, control diet; HLD, high lard diet; HOD, high olive oil diet; CO, cardiac output; ESP, end-systolic pressure; EDP, end-diastolic pressure; ESPVR, end-systolic pressure volume relationship; EDPVR, end-diastolic pressure-volume relationship; EF, ejection fraction; FS, fractinal shortening; IVS, interventricular septum thickness during diastole; LV, left ventricular; LVEDD, left ventricular end-diastolic diameter; LVESD, left ventricular end-diastolic diameter; PW, posterior wall thickness; Data were presented as mean ± SEM.

* *P* < 0.05 vs CD,

** *P* < 0.01 vs CD,

^#^ *P* < 0.05 vs HLD,

^##^ *P* < 0.01 vs HLD, by ANOVA followed by post hoc Tukey–Kramer tests.

**Fig 2:**
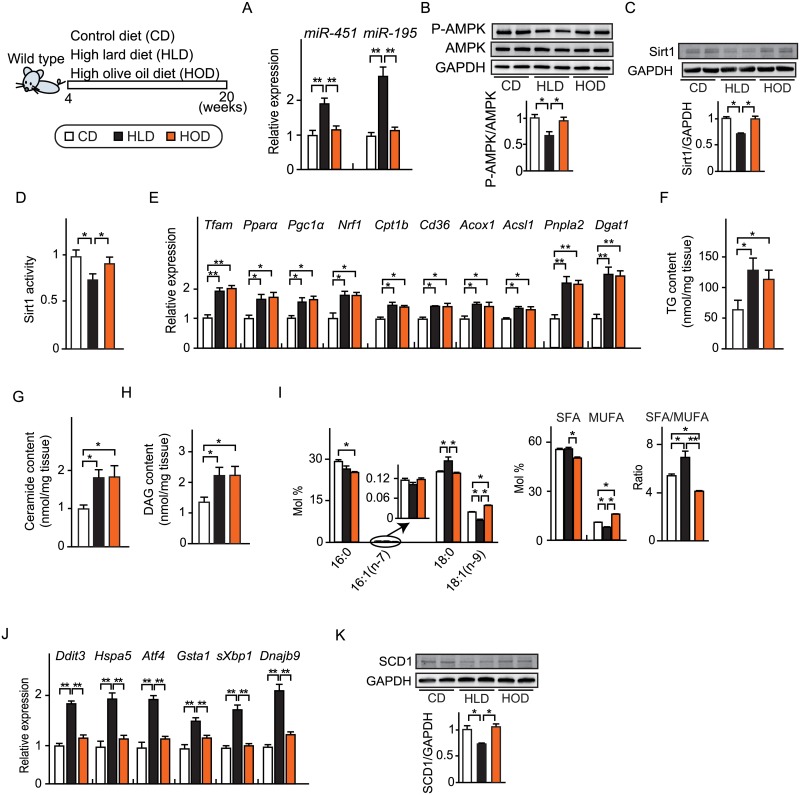
SFA-rich HFD increased fatty acid saturation in cardiac membrane phospholipids. (A) Relative microRNA (miR) expression. (n = 6). (B) Top, Representative immunoblots of phospho-AMPK(Thr172)(P-AMPK), AMPK and GAPDH. Bottom, Quantification of P-AMPK to AMPK ratio by densitometry analysis. (n = 3). (C) Top, representative immunoblots of Sirt1 and GAPDH. Bottom, quantification of Sirt1 by densitometry analysis. (n = 4). (D) Relative Sirt1 deacetylase activity. (n = 6). (E) Relative gene expression of mitochondrial biogenesis, TG turnover, PPARα-target genes in hearts. (n = 6). (F) Triglyceride (TG) content in hearts. (n = 4). (G) Ceramide content in hearts. (n = 4). (H) Diacylglycerol (DAG) content in hearts. (n = 4). (I) FA composition (mol% of phospholipids) of saturated fatty acid (SFA), monounsaturated fatty acid (MUFA) and the SFA/MUFA ratio. (n = 4). (J) Relative expression of UPR signaling genes. (n = 5). sXBP1 indicates spliced-XBP1. (K) Top, representative immunoblots of SCD1 and GAPDH. Bottom, quantification of SCD1 by densitometry analysis. (n = 4). Data were presented as mean ± SEM. * *P* < 0.05, ** *P* < 0.01, by ANOVA followed by post hoc Tukey–Kramer tests. See also Table 1.

We compared the lipid metabolism in the heart between the HLD and HOD groups. It has been shown that diabetic conditions up-regulates miR-451 and miR-195 and suppresses protein expression of AMPK and Sirt1 and their activities, respectively [36, 37]. Interestingly, HLD, but not HOD, upregulated miR-451 and miR-195 expression in the heart (Fig 2A). Accordingly, HLD, but not HOD decreased AMPK activity and protein expression of Sirt1 and its activity in the heart (Fig 2B–2D). Both HLD and HOD increased the expression of the transcriptional regulators of mitochondrial function (*Tfam*, *Pparα*, *Pgc1α*, *Nrf1*) and genes involved in TG turnover (*Pnpla2*/*Atgl*, *Dgat1*) compared to the CD group (Fig 2E). However, there was no difference in the degree of change in this gene expression between the HLD and HOD groups. Similarly, both HLD and HOD increased the PPARα-target genes involved in fatty acid uptake and mitochondrial oxidation (*Cpt1*, *Cd36*, *Acox1*, *Acsl1*) to the same extent. As predicted by these results, both HLD and HOD caused comparable accumulation of TG, diacylglycerol (DAG), and ceramide in the heart (Fig 2F–2H).

By contrast, fatty acid composition of membrane phospholipids in the heart was differentially influenced by HLD and HOD. The HLD raised the proportion of stearate (C18:0) and lowered the proportion of oleate (C18:1), thereby increasing the SFA/MUFA ratio (= membrane fatty acid saturation). In parallel to increase in SFA/MUFA ratio in membrane phospholipids, the HLD induced UPR (Fig 2I and 2J). By contrast, the HOD lowered the proportion of palmitate (C16:0) and raised the proportion of oleate (C18:1), thereby lowering the SFA/MUFA ratio. Consistent with this, HOD did not induce UPR. The expression of SCD1, an integral membrane protein anchored in the ER and a rate-limiting enzyme in the cellular synthesis of MUFA from SFA, was significantly decreased in the HLD group, but not in the HOD group (Fig 2K).

Taken together, we demonstrated that when mice were fed a HFD, excess SFA from the diet directly increases SFA content in membrane phospholipids and at the same time decreased the expression/activity of SCD1 determining the SFA/MUFA balance in membrane phospholipids. The differential impact of HLD and HOD on LV remodeling and diastolic dysfunction is probably not explained by the differential ability of SFA and MUFA to activate PPARα signaling, TG turnover, and energy production in mitochondria. Rather, the severity of LV remodeling and diastolic dysfunction was correlated with the degree of FA saturation (an increase in SFA content) in the membrane phospholipids.

### Exogenously given saturated fatty acid directly increased the degree of fatty acid saturation in membrane phospholipids in cultured neonatal rat cardiomyocytes

**Fig 3:**
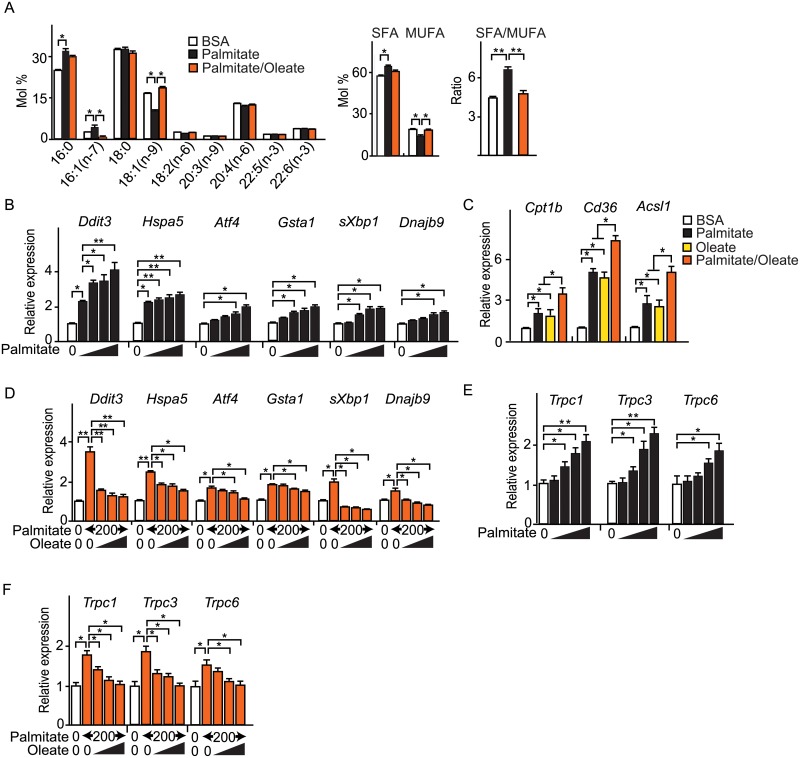
Palmitate increased SFA/MUFA ratio in membrane phospholipid and induced UPR in neonatal rat cardiomyocytes. Neonatal rat cardiomyocytes were stimulated by fatty acids (200 μmol/L). (A) Fatty acid composition (mol% of phospholipids) of saturated fatty acid (SFA), monounsaturated fatty acid (MUFA) and the SFA/MUFA ratio after fatty acid stimulation for 6 hours. (n = 4). (B) Relative expression of UPR signaling genes after palmitate stimulation for 6 hours. Concentrations of palmitate were 50, 100, 200 and 400 μmol/L. (n = 4). sXBP1 indicates spliced-XBP1. (C) Relative expression of PPARα-target genes after co-administration of palmitate and oleate for 6 hours. Concentrations of each fatty acid was 200 μmol/L. (n = 4). (D) Relative expression of UPR signaling genes after co-administration of palmitate and oleate for 6 hours. Concentrations of oleate were 50, 100 and 200 μmol/L. (n = 4). sXBP1 indicates spliced-XBP1. (E) Relative expression of TRPC channels after palmitate stimulation for 12 hours. Concentrations of palmitate were 50, 100, 200 and 400 μmol/L. (n = 4). (F) Relative expression of TRPC channels after co-administration of palmitate and oleate for 12 hours. Concentrations of oleate were 50, 100 and 200 μmol/L. (n = 4). Data were presented as mean ± SEM. * *P* < 0.05, ** *P* < 0.01, by ANOVA followed by post hoc Tukey–Kramer tests.

To determine whether exogenous SFA from the diet directly increases SFA content in membrane phospholipids, we examined a change in the FA composition of membrane phospholipid by adding different FA to culture media. Palmitate increased the ratio of SFA to MUFA in membrane phospholipid by increasing the proportion of palmitate (C16:0) and lowering the proportion of oleate (C18:1). When given in concert, oleate canceled the palmitate-induced changes in the SFA/MUFA ratio by restoring the proportion of oleate (Fig 3A). Representative SFA, palmitate induced UPR by dose dependent manner (Fig 3B). These findings indicate that the SFA/MUFA ratio in cardiomyocyte membrane phospholipid is easily affected by exogenously given fatty acids. Palmitate and oleate (representative MUFA) increased the expression of PPARα-target genes (*Cpt1*, *Cd36*, *Acsl1*) to the same extent, and co-administration of palmitate and oleate caused additive increase in the expression of PPARα-target genes (Fig 3C). Palmitate-induced UPR were canceled by co-administration of oleate by dose dependent manner (Fig 3D). Likewise, co-administration of oleate canceled the palmitate-induced TRPC channels genes-upregulation by dose dependent manner (Fig 3E and 3F). These findings suggest that UPR occurs when the SFA/MUFA ratio in cardiomyocyte membrane phospholipid increases.

These results indicate that the surplus of SFA in the culture media is channeled into membrane phospholipids, leading to an increase in SFA/MUFA ratio of the membrane phospholipid in cardiomyocytes. SFA-induced upregulation of TRPC channels are directly linked to an increase in SFA/MUFA ratio of the membrane phospholipid in cardiomyocytes.

## Discussion

The adult cardiomyocytes in actively contracting and load-bearing myocardium exhibit the high ATP demand of cells with high FA oxidation rates and capacity, with high FAO enzyme expression and mitochondrial density. It seems unlikely that such adult cardiomyocytes in situ would channel a substantial amount of exogenous FA toward lipid membranes, unlike other cell types that have no incentive to burn fat. In this study, we found that when FA overload in the heart overwhelms the cellular capacity to use it for energy, the surplus of FA is channeled into membrane phospholipids. In such situations, the FA composition of membrane phospholipids in adult cardiomyocytes is directly affected by exogenous FA from the diet.

Despite the fact that 16 weeks of an HLD (SFA-rich HFD) and an HOD (MUFA-rich HFD) caused the same degree of visceral obesity, the former resulted in more severe pathological cardiac hypertrophy, fibrosis, and diastolic dysfunction than the latter. However, after 16 weeks of feeding, no differences were observed in the expression levels of transcriptional regulators of mitochondrial function, genes involved in TG turnover, myocardial FA uptake, and mitochondrial FA oxidation, known as PPARα-target genes. Furthermore, there were no difference in terms of accumulation of toxic lipid intermediates, such as ceramide and DAG, in the heart between mice chronically fed an HLD and an HOD. Based on these observations, we concluded that the differences in hypertrophic remodeling and diastolic dysfunction between the HLD-fed hearts and the HOD-fed hearts is unlikely to be explained by differential activation of PPARα-signaling and the accumulation of toxic lipid intermediates. The difference between the two was, rather, that an HLD decreased the membrane phospholipid unsaturation (raised the SFA/MUFA ratio of the membrane phospholipids) in the heart, whereas an HOD did not. An HLD, but not an HOD, decreased the expression of SCD1, a rate-limiting enzyme in the cellular synthesis of MUFA from SFA which determines the SFA/MUFA balance in membrane phospholipids. Taken together, we concluded that dietary fat quality (the SFA/MUFA ratio) does not affect the degree of activation of PPARα in the heart during the taking of excess fat. Rather, dietary fat quality affects the SFA/MUFA ratio of the membrane phospholipid composition of FA in cardiomyocytes. Increased saturation of membrane phospholipids correlates with the worsening of diastolic parameters.

Proof for the link between the decrease in membrane phospholipid unsaturation and LV diastolic dysfunction must await future experiments. However, we thought that SFA trafficking to the ER membrane and subsequent change in ER membrane fluidity are proximal to this connection based on the following observations. The ER membrane has a relatively low free cholesterol, phospholipid ratio, and SFA content in the FA composition of phospholipids, and it is, therefore, one of the most fluid membranes in the cell [38, 39]. A number of integral proteins in the ER membrane are adapted to function optimally in this fluid membrane environment and would therefore be adversely affected by an increase in membrane stiffness. Sarcoplasmic reticulum calcium ATPase (SERCA) contains 11 membrane-spanning regions and calcium pumping cycle requiring several changes in protein conformation [40]; in vitro experiments showed that SERCA activity is compromised when the membrane order (stiffness) increased after incubation with phosphatidylcholine containing SFA [41]. Dysregulation of Ca^2+^ handling gene expression was often observed in animal models of diastolic dysfunction [42]. A decrease in the rate of Ca^2+^ reuptake via SERCA2a results in a state of Ca^2+^ overload. This leads to a slow or incomplete relaxation of the ventricles, causing diastolic dysfunction [43]. Probably, SERCA2a loses its function due to restriction of the conformation changes in a more ordered SFA-enriched membrane.

Interestingly, the expression of Trpc1, Trpc3, Trpc6 were upregulated in heart of HLD-fed mice, but not in heart of HOD-fed mice. In culture, palmitate induced expression of Trpc1, Trpc3, Trpc6 and co-administration of oleate canceled palmitate-induced TRPC channels genes. Enhanced Ca^2+^ reentry through TRPC channels are established upstream regulator of diastolic dysfunction associated with hypertrophic remodeling [44–46]. TRPC promotes cardiomyocyte hypertrophy through activation of calcineurin and its downstream effector, the nuclear factor of activated T cells (NFAT) transcription factor [47]. These biophysical models could be a key upstream event underlying the link between decrease in membrane phospholipid unsaturation, induction of UPR, and development and progression of diastolic dysfunction in SFA-overfed hearts.

## Supporting information

S1 Fig Different effects on HLD and HOD in mice. Serum fatty acid composition (n = 4), body weight (n = 10), epididymal fat weight (n = 6), liver weight (n = 6), oral glucose tolerance test (n = 5), insulin tolerance test (n = 5) of mice fed either CD, HLD or HOD and data were presented as mean ± SEM, * *P* < 0.05, ** *P* < 0.01, *#P* < 0.05 vs HOD by ANOVA followed by post hoc Tukey–Kramer tests.
(PDF)

S1 Table Primer sequences for mouse. (PDF)

S2 Table Primer sequences and assay ID for rat. (PDF)
