# Supplementary material for: Decrease in membrane phospholipids unsaturation correlates with myocardial diastolic dysfunction
Source: PLoS One. 2018 Dec 11;13(12):e0208396. doi: 10.1371/journal.pone.0208396 (PMC6289418; doi:10.1371/journal.pone.0208396)
Supplement: S1 Fig — Serum fatty acid composition (n = 4), body weight (n = 10), epididymal fat weight (n = 6), liver weight (n = 6), oral glucose tolerance test (n = 5), insulin tolerance test (n = 5) of mice fed either CD, HLD or HOD and data were presented as mean ± SEM, * P < 0.05, ** P < 0.01, #P < 0.05 vs HOD by ANOVA followed by post hoc Tukey–Kramer tests. (PDF) [file pone.0208396.s001.pdf]

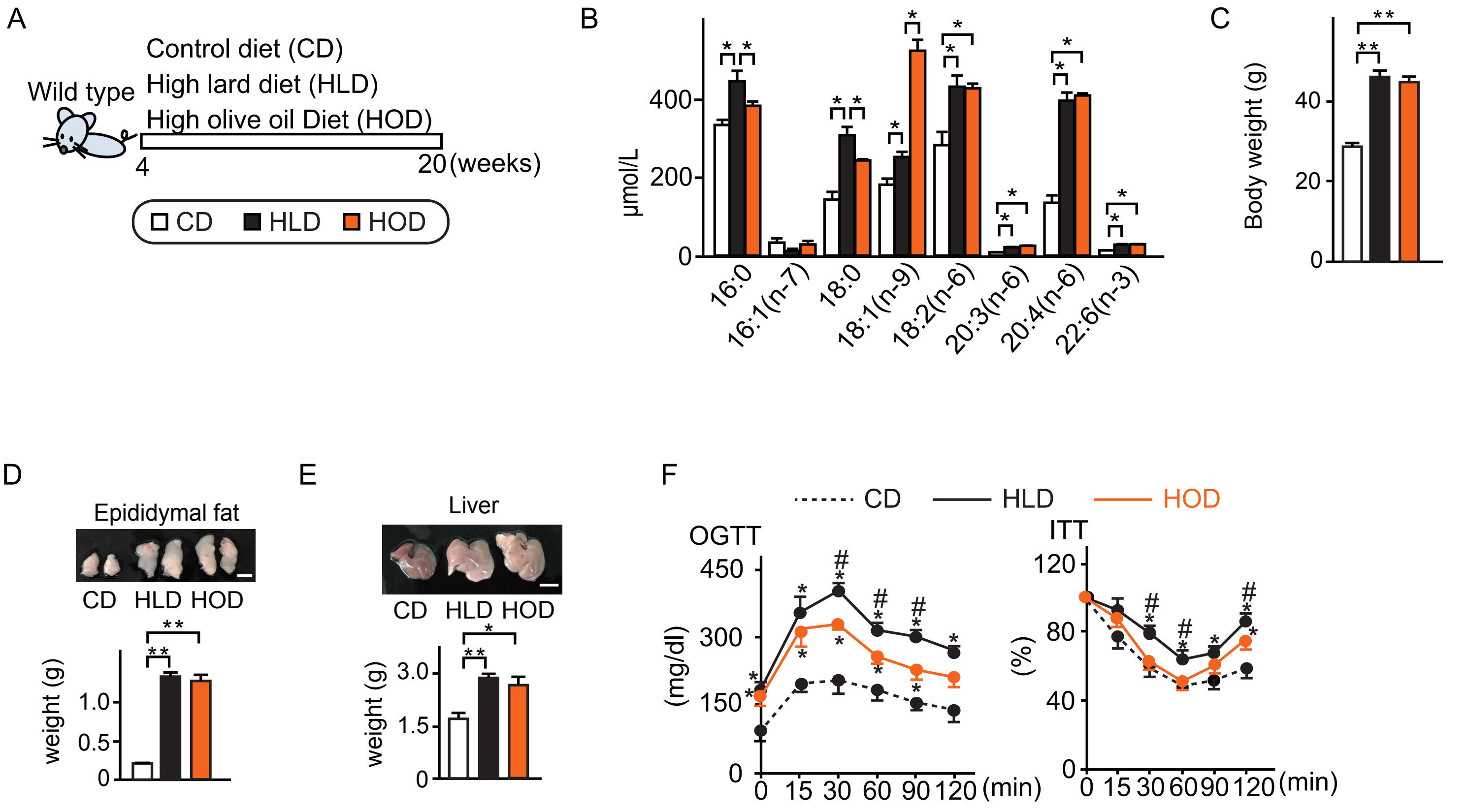

S1 Fig. Different effects on HLD and HOD in mice. Serum fatty acid composition (n=4), body weight (n=10), epididymal fat weight (n=6), liver weight (n=6), oral glucose tolerance test (n=5), insulin tolerance test (n=5) of mice fed either CD, HLD or HOD and data were presented as mean ± SEM, *P < 0.05, **P < 0.01, #P < 0.05 vs HOD by ANOVA followed by post hoc Turkey-Kramer tests.
