# Supplementary material for: Decrease in membrane phospholipids unsaturation correlates with myocardial diastolic dysfunction
Source: PLoS One. 2018 Dec 11;13(12):e0208396. doi: 10.1371/journal.pone.0208396 (PMC6289418; doi:10.1371/journal.pone.0208396)
Supplement: S1 Table — (PDF) [file pone.0208396.s002.pdf]

S1 Table. Primer sequences for mouse.

Target gene	Forward (5’ -3’)	Reverse (5’ -3’)
Gapdh	TCAACGGCACAGTCAAGG	CACGACATACTCAGCACC
Myh7	CCTGCGGAAGTCTGAGAAGG	CTCGGGACACGATCTTGGC
Myh6	GCCCAGTACCTCCGAAAGTC	ATCAGGCACGAAGCACTCC
Tgfb1	CCACCTGCAAGACCATCGAC	CTGGCGAGCCTTAGTTTGGAC
Col1a1	GCTCCTCTTAGGGGCCACT	ATTGGGGACCCTTAGGCCAT
Cpt1b	GACTTCCGGCTTAGTCGGG	GAATAAGGCGTTTCTCCAGGA
Cd36	ATGGGCTGTGATCGGAACTG	TTTGCCACGTCATTCTGGGTTT
Acox1	TAACTTCCTCACTCGAAGCCA	AGTTCCATGACCCATCTCTGTC
Acsl1	TGCCAGAGCTGATTGACATTC	GGCATACCAGAAGGTGGTGAG
Ddit3	AAGCCTGGTATGAGGTCGC	TTCCTGGGGATGAGATAGGTG
Hspa5	ACTTGGGGACCACCTATTCCT	GTTGCCCTGATCGTTGGCTA
spliced XBP1	TGAGAACCAGGAGTTAAG	CCTGCACCTGCTGCGGAC
Dnajb9	TCAGAGCGACAAATCAAAAAGGC	CTATTGGCATCCGAGAGTGTTT
Atf4	CCTGAACAGCGAAGTGTTGG	TGGAGAACCCATGAGGTTTCAA
Gsta1	AAGCCCGTGCTTCACTACTTC	GGGCACTTGGTCAAACATCAAA
Tfam	AACACCCAGATGCAAAACTTTC	GACTTGGAGTTAGCTGCTCTTT
Pparα	GGAAGACCACTCGCATTCCTT	GTAATCAGCAACCATTGGGTCA
Pgc1α	TATGGAGTGACATAGAGTGTGC	GTCGCTACACCACTTCAATCC
Nrf1	AGCACGGAGTGACCCAAAC	AGGATGTCCGAGTCATCATAAG
Pnpla2	ATGTTCCCGAGGGAGACCAA	GAGGCTCCGTAGATGTGAGTG
Dgat1	GTGCCATCGTCTGCAAGATTC	GCATCACCACACACCAATTCAG
Trpc1	GATGTGCTTGGGAGAAATGCT	ACTGACAACCGTAGTCCAAAAG
Trpc3	GCCTTCATGTTCGGTGCTC	GGTCACCTCCAGATGCTCATT
Trpc6	GGCGGCTCTCTAAAGGCTG	TGGGGTAGTAGCCATACGGTG
