# Supplementary material for: Decrease in membrane phospholipids unsaturation correlates with myocardial diastolic dysfunction
Source: PLoS One. 2018 Dec 11;13(12):e0208396. doi: 10.1371/journal.pone.0208396 (PMC6289418; doi:10.1371/journal.pone.0208396)
Supplement: S2 Table — (PDF) [file pone.0208396.s003.pdf]

S2 Table. Primer sequences and assay ID for rat.

Target gene	Forward (5’ -3’)	Reverse (5’ -3’)
Gapdh	GGCAAGTTCAATGGCACAGT	TGGTGAAGACGCCAGTAGACTC
spliced XBP1	TGAGAACCAGGAGTTAAG	CCTGCACCTGCTGCGGAC
Trpc1	TATGGGGAAGAACTGCAGTCC	CAGATCTTGGCGCAGTTCATT
Trpc3	CGTTCCAAACTCTGGCTATCC	AAGGCTGGAGATATCCTGCTT
Trpc6	CTTGTGCCAAGTCCAAAGTCC	TTCCTTCAGCTCCCCTTCGTT

Target gene	Taqman gene expression assay number
Gapdh	Rn01775763_g1
Ddit3	Rn00492098_g1
Hspa5	Rn00565250_m1
Atf4	Rn00824644_g1
Gsta1	Rn00580416_m1
Dnajb9	Rn01473728_m1
Cpt1b	Rn00682395_m1
Cd36	Rn02115479_g1
Acsl1	Rn00563137_m1
Atp2a2	Rn00568762_m1
